# Fighting against a protean enemy: immunosenescence, vaccines, and healthy aging

**DOI:** 10.1038/s41514-017-0020-0

**Published:** 2017-12-21

**Authors:** Giuseppe Del Giudice, Jörg J. Goronzy, Beatrix Grubeck-Loebenstein, Paul-Henri Lambert, Tomas Mrkvan, Jeffrey J. Stoddard, T. Mark Doherty

**Affiliations:** 1 GSK, Siena, Italy; 2 Stanford University School of Medicine, Stanford, CA USA; 3 University of Innsbruck, Innsbruck, Austria; 4 University of Geneva, Geneva, Switzerland; 5 GSK, Wavre, Belgium; 6 GSK, Philadelphia, PA USA

## Abstract

The progressive increase of the aged population worldwide mandates new strategies to ensure sustained health and well-being with age. The development of better and/or new vaccines against pathogens that affect older adults is one pivotal intervention in approaching this goal. However, the functional decline of various physiological systems, including the immune system, requires novel approaches to counteract immunosenescence. Although important progress has been made in understanding the mechanisms underlying the age-related decline of the immune response to infections and vaccinations, knowledge gaps remain, both in the areas of basic and translational research. In particular, it will be important to better understand how environmental factors, such as diet, physical activity, co-morbidities, and pharmacological treatments, delay or contribute to the decline of the capability of the aging immune system to appropriately respond to infectious diseases and vaccination. Recent findings suggest that successful approaches specifically targeted to the older population can be developed, such as the high-dose and adjuvanted vaccines against seasonal influenza, the adjuvanted subunit vaccine against herpes zoster, as well as experimental interventions with immune-potentiators or immunostimulants. Learning from these first successes may pave the way to developing novel and improved vaccines for the older adults and immunocompromised. With an integrated, holistic vaccination strategy, society will offer the opportunity for an improved quality of life to the segment of the population that is going to increase most significantly in numbers and proportion over future decades.

## Introduction

Global population changes pose serious challenges to health systems worldwide. Global reductions in birth rates, and reduced mortality have increased life expectancy in both the developed and developing world.^1^ The associated shift in age demographics requires new approaches to ensure that increased life spans do not come at the expense of an optimal quality of life for older adults. New approaches need to take into consideration the deteriorating effect of age on organ and system function, and the intrinsic and extrinsic (environmental) factors that influence the development of this deterioration.

**Fig. 1:**
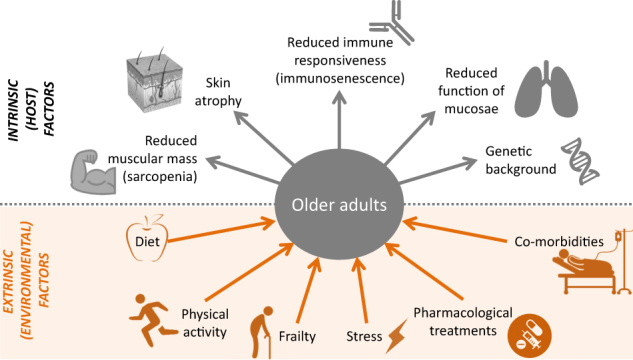
Factors influencing immunosenescence. Aging is influenced by multifaceted extrinsic and intrinsic factors leading to a progressive impairment of the function of various organs and systems, including the immune responsiveness, leading to increased susceptibility to infectious diseases and to reduced response to vaccination

Aging is characterized by multifaceted changes in the immune system which lead to a progressive reduction of the ability to mount effective antibody and cellular responses against infections and to vaccinations. This phenomenon, referred to as immunosenescence, is multifactorial: it affects both arms of the immune system and can be influenced by genetic factors and extrinsic factors, such as nutrition, physical exercise, co-morbidities, physical and mental stress, previous exposure to microorganisms, toxins, and pharmacological treatments (Fig. 1).^2^ Consequently, the presenting forms of immunosenescence are protean, varying at population and individual levels. Therefore the concept of “Bioage” is arising to describe the concept that the real age is not the chronological, but the biological one.

Understanding the contributions of these intrinsic and extrinsic factors can unravel the mechanisms leading to the progressive waning of immune competence with age and hopefully identify the means to delay or counteract it.

Vaccines represent one of the most powerful medical interventions against infectious diseases.^3^ National vaccine programs have been extremely successful in reducing mortality and morbidity worldwide: This reduction can reach 100%, evident in the case of smallpox, with poliomyelitis getting closer and closer to global eradication, and regional elimination, in the case of measles.^4,5^ However, current vaccination programs are mainly focused in reducing morbidity and mortality in children.^6^ The same attention is rarely paid to adult vaccination programs. Effective adult vaccination programs targeting all age groups, including older adults are now more urgent than ever. Changing demographics and the vast increase of aged individuals, necessitates development of efficacious and safe vaccines, suitable for adults and in particular for older adults. Some existing vaccines are recommended for use in older adults, for example those against influenza, herpes zoster, diphtheria, tetanus, pertussis, hepatitis and pneumococcal disease.^7^ However, their efficacy and coverage may be sub-optimal, especially in the oldest segment of the population.

Recent progress in immunology, molecular biology, and systems biology is solving many of the biological problems that characterize the aging immune response.^8^ However, immunological changes are often studied in isolation, without an integrated overview. The problem of an aging immune response demands interdisciplinary approaches between scientists, epidemiologists, clinicians, and public health decision-makers. Such an approach can create a conceptual framework to facilitate the development of more appropriate vaccines and strategies to effectively protect aging populations. An important target is infectious diseases, not only for the increased burden and risk in this age category but because this may potentially reduce the burden of some non-infectious diseases, such as cancer, cardiac and cerebrovascular disease, etc.^9,10^ thereby improving both life expectancy and quality of life of older persons.

## Benefits of vaccination/immunostimulation in older adults

Infections, especially those of the respiratory tract, and their complications are a significant cause of death across all age groups worldwide. Morbidity and mortality due to respiratory infections such as influenza, pneumococci, respiratory syncytial virus (RSV), and pertussis increase in subjects ≥65-years old.^11^ The risk of herpes zoster increases with age, and is particularly high in ≥65-year-olds.^12^ Finally, increasing incidences of infection due to diseases such as *Clostridium difficile*, vancomycin resistant enterococci, Group B streptococci, and antibiotic resistant *Staphylococcus aureus* are being reported worldwide in ageing people.^13^ Infection in older adults can exhibit clinical manifestations different from those typically observed in younger subjects in terms of signs, symptoms, progression and outcome, as seen, for example, in bacterial meningitis.^14^

While vaccines targeting older populations exist, their performance is often sub-optimal and/or they are under-used. Vaccination against influenza has proved effective in reducing morbidity and mortality in older adults, although its performance is less than ideal and lower than that observed in younger adults, especially against drifted A virus strains.^15^ Vaccine efficacy against hepatitis B virus (HBV) and A virus (HAV), tick-borne encephalitis (TBE), is significantly reduced in vaccinated subjects ≥70–80-year-old; in the case of HBV and HAV vaccines for example, obesity and smoking were additional factors impacting vaccine response in adults and older adults.^16^ Similarly, efficacy of the live attenuated vaccine against herpes zoster is progressively reduced with increasing age.^17^

At present, major gaps exist in our knowledge of the mechanisms behind the reduced ability of the aging immune system to respond appropriately to both infections and vaccinations. This hinders our ability to design interventions capable of improving the immune response in older adults and to tailor vaccines better suited for this group.

## The protean faces of immunosenescence

**Fig. 2:**
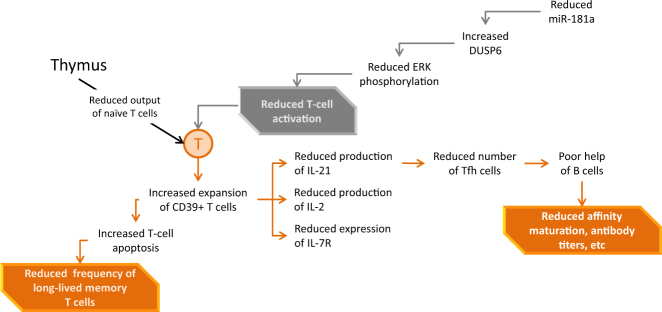
T-cell changes associated with age. DUSP6 dual specificity phosphatase 6, miR-181a, microRNA-181a, ERK extracellular signal-regulated kinases, Tfh T follicular helper

Immunosenescence is a multifactorial phenomenon that affects all compartments of the immune system. T cells are dramatically affected. The drastic change of the thymus, which involutes and atrophies with age, results in the failure to generate new pluripotent T cells and the contraction and aging of the naïve T-cell compartment.^18^ T-cell functions are also affected, with defects experienced at the level of T-cell activation, memory and signaling, clonal expansion and development of antigen-specific effector cells, and of long-lived memory T cells. The T-cell receptor (TCR) repertoire breadth in 70–85-year-olds is only 10–25% of that found in 20–35-year-olds.^19^ Nevertheless, this reduced repertoire is still extremely diverse and likely sufficient to achieve protection, if appropriately activated. Indeed, following vaccination with a live attenuated vaccine against herpes zoster, despite a decrease in antigen-specific T cell helper CD4^+^ cells, a rich and diverse TCR repertoire is still available with varicella zoster virus (VZV)-specific CD4+ clones recruited from the naïve T-cell pool.^20^ Changes in responsive T-cell availability are accompanied in >70-year-olds with functional defects including reduced signaling via the TCR. An important underlying defect is the age-associated decline in the expression of micro RNA-181a (miR181a) and an associated increase of its target DUSP6 (Dual specificity phosphatase 6) that results in defective phosphorylation of the extracellular signal-regulated kinases (ERK) necessary for T-cell clonal expansion and effector differentiation.^21^ Finally, the propensity of effector T cells to survive and differentiate into long-lived memory cells is determined by ecto-ATPase CD39 expression and by purinergic signaling. CD39 expression is increased in the CD4+ T cells of older individuals, leading to increased loss of antigen-specific T cells by apoptosis^22^ (Fig. 2). Changes are even more evident in CD8+ T cell populations which tend to lose the co-stimulatory molecule CD28, live longer, proliferate less and exhibit reduced TCR diversity and functional capacities.^23^ Characterization of molecular defects is a first step to identify targets for possible immune potentiating therapies in vaccine responses.

These changes can lead to a reduced homeostatic control of chronic or latent infections, such as VZV which can reactivate, causing herpes zoster (shingles), a disease particularly frequent among older adults and immune-compromised individuals due to treatment or concomitant infections, such as human immunodeficiency virus (HIV).

While initial events in T-cell aging occur in secondary lymphoid structures in the absence of a thymus, homeostatic proliferation accounts for T-cell generation, additional key events also take place with age in the bone marrow. These events affect all compartments of the immune system, involving adaptive and innate immunity. Pro-inflammatory cytokines such as IL-15 and IL-6 increase in the bone marrow of older adults, along with CD8+ CD28- accumulation and CD8+CD69+ T cells which produce interferon-gamma (IFN-γ) and tumor necrosis factor-alpha (TNF-α), plus other pro-inflammatory cytokines.^23^^,^^24^ Concomitantly, there is a significant reduction of total and antigen-specific plasma cells with age, for example those against tetanus and diphtheria. This is not the case for some diseases such as influenza and cytomegalovirus (CMV),^25^ probably due to persistent chronic CMV or repeated exogenous stimulation via vaccinations, sub-clinical or clinically overt infections.

Pro-inflammatory environments and the reduction of hematopoietic tissue in the bone marrow with age lead to lower production of pro-B lymphocytes and reduced differentiation into pre-B and into mature B lymphocytes, the latter being able to leave the bone marrow. This results in a reduced number of circulating naïve B cells and a concomitant relative accumulation of memory B cells,^26^^,^^27^ which, as in case of the TCR, express a limited diversity in the B-cell antigen receptor (BCR) repertoire.^28^^,^^29^ The final outcome of this dysregulation translates into a reduced ability of aged B cells to mount an appropriate antibody response against new or known antigens, where the antibody response is short-lived with defects in isotype switching.^30^^,^^31^ Reduced isotype switching efficiency in older adults is associated with reduced expression of activation-induced cytidine deaminase (AID), which has been proposed as a potential biomarker predicting the response to antigens following vaccination in both young and older adults.^32^ The level of switched memory B cells (CD27+ IgD-B cells), which are significantly reduced in the older adults, has recently been shown to predict the responsive ability of almost 80% of older adults to influenza vaccination.^33^ These biomarkers may turn out to be particularly useful at two levels: (i) predicting the ability of older subjects to respond to vaccination, and (ii) defining target molecules whose expression/levels could be regulated by better vaccines designed for this population.

Aging affects innate immunity to a lesser extent than adaptive immunity. Many observed changes are due to lower expression of toll-like receptors (TLR), notably TLR1 on monocytes and neutrophils, which are less metabolically active (reduced glycolysis) upon inflammatory stimuli.^34^ This can translate into reduced anti-bacterial activities of neutrophils and monocytes and reduced ability of macrophages to activate adaptive immune responses. The dysregulation of TLR3 expression and changes in the natural killer (NK) cell subpopulations in the older adults has been associated with a lower response to West Nile Virus in vitro a disease to which older adults are particularly susceptible.^35^^,^^36^

Up to now however no definitive biomarkers of immunosenescence have been identified. A big effort towards this end is being made in Europe through collaborative networks of scientists. One example is the MARK-AGE project which conducted population studies with about 3200 subjects to identify potential biomarkers of ageing.^37^ Other similar initiatives are being undertaken in Europe, USA and Asia. However, the difficulty of defining biomarkers of ageing may be linked to the intrinsic heterogeneity of aging populations, underlining the importance of biological age over chronological age, and the necessity of an holistic approach taking into consideration all the factors influencing the waning of the response to infections and vaccination.

## Integrating the elements: the systems biology approach

Recent progress in the fields of immunology, molecular biology and systems biology have enabled improved understanding of the innate and adaptive immunity mechanisms behind the sub-optimal responsiveness of the older adults to vaccination. These methodologies are now being applied to various vaccination strategies for older adults to identify biomarkers which may predict low vaccine responsiveness and help design vaccines tailored for this population. In addition, it is becoming increasingly evident that older individuals should not be conceptualized as isolated biological entities. Instead, their health status and ability to respond to infections and vaccination is influenced by extrinsic, contextual environmental factors linked to individual variables, such as nutrition, physical exercise, drug treatments, co-morbidities, etc.

A comprehensive approach to understanding the quality of antibody response with age can derive from the next generation high-throughput sequencing of the variable regions of antigen-specific immunoglobulins, derived from B cells or circulating plasmablasts. Information on how this B cell response translates into biologically relevant antibody quantities comes from proteomic studies of the repertoire of circulating antibodies, which are now being successfully applied to dissect immune response to influenza vaccination.^38^

A study conducted in older adults who received HBV vaccine has shown that some markers of hyporesponsiveness can be identified before vaccination. These markers, evidenced at the level of mRNA transcripts, were also confirmed phenotypically by cytometric profiling. While increased expression of genes related to B-cell responses and memory B-cell frequencies was associated with strong responses to HBV vaccine, a weaker antibody response was associated with increased expression of genes linked to pro-inflammatory responses and innate cells.^39^ This study also clearly showed that the association with lower responsiveness was not exclusively linked to the chronological age, since some “younger” subjects exhibited the same pattern of hyporesponsiveness while other, “older”, subjects were relatively good responders. These findings introduce the concept of “bio-age” underlining the importance of biological changes over chronological changes. Furthermore, this study also showed an association between weaker antibody responses and higher expression of genes linked with erythropoietin expression and the number of circulating red blood cells. It is conceivable that this may be due to the inflammatory events present in the aging body which lead to tissue hypoxia and consequent increase of red blood cells counts and the expression of genes regulating their production. The concept of “bio-age” is in line with the observation of the wide variability of immune responses observed in the elderly after vaccination. In addition, it is in line with the increasing evidence that the immunological experience that individuals have during their lives can shape their ability to respond to external stimuli, such as infections or vaccinations. This concept is now being defined as “immunobiography”.^40^ It encompasses various events both intrinsic to the aging organism and extrinsic, including nutrition factors, physical exercise, drug treatments (see below), microbiota, etc. These factors could not only affect the adaptive immune response to infections and vaccination, but also those elements of the innate immunity which are referred to as trained immunity.^41^ These elements include macrophages, various kinds of NK cell populations, innate lymphoid cells, γδ T cells, and can exhibit features of immunological memory which appear to be of epigenetic nature. It is striking to note that some of the pro-inflammatory events which characterize both inflamm-aging and trained immunity (e.g. macrophage, NK cells, secretion of cytokines, etc.) are those which are rapidly activated by some vaccine adjuvants, and which may promote mechanism(s) of action leading to the beneficial effects of these adjuvanted vaccines(see below).

In another study, older adults vaccinated with seasonal trivalent influenza vaccine (TIV) displayed a baseline signature of lymphocyte inflammation which positively correlated with the post-vaccination antibody response, while the reverse occurred with a monocyte inflammation signature at baseline.^42^ In contrast, such baseline signatures at the levels of predictive signatures were not evident in a study with older adults vaccinated with live-attenuated vaccine against herpes zoster.^43^ These observed differences could be explained by heterogeneity in the population studies or to the intrinsic differences among vaccines (inactivated vs. live; unadjuvanted vs. adjuvanted, etc.) which could trigger different pathways at different time points.

Recent studies have highlighted the need for a systems biology approach, for example, integrating mRNA sequencing with data derived from analysis of peripheral blood cells, and DNA methylation. Only in this way was it possible to predict the antibody response to vaccination against influenza.^44^

The pro-inflammatory environment of the aging body is a common denominator of aging which is referred to as “inflamm-aging”.^45^ This refers to the pleiotropic, multifactorial inflammatory events which characterize an aging body. Extensive scientific literature has been published on this area and among the many hypothetical potential causes, the most popular is infection with CMV. Indeed, strong CMV seropositivity has been associated with lower antibody and cellular responses to a variety of vaccines, including influenza, diphtheria, tetanus, etc.^46^^,^^47^ The long-term maintenance of vaccine-specific antibodies seems to be hampered by CMV, as antibodies against diphtheria are decreased 5 years after vaccination in older persons with positive CMV serology (unpublished data). Studies have shown that CMV seropositivity in healthy adults correlated with an up-regulation of immune components, including increased antibody responses to influenza vaccine, CD8+ T cell level and circulating IFN-γ as compared to non-infected subjects, while the difference between CMV-infected and uninfected individuals was less visible in older adults.^48^

A longitudinal systems immunology study compared immunological status (serum cytokines and antibodies, and reactivity/signaling pathways in blood leukocytes) in children, young adults, and older adults, and a twin study has shown that vaccination response appears to be heavily influenced by environmental exposure.^49^ This is particularly important in older adults, in which exposure history seems to be more important than an individual’s genetic background.

A longitudinal genome-wide association study (GWAS) was carried out in Sardinia in a cohort of 1629 individuals from four clustered Sardinian villages aged 10–105-years, evaluating the relative role of genetic background vs. environmental factors in determining immune phenotypical and functional features.^50^ Genetic contribution to quantitative levels of 95 cell types encompassing 272 immune traits was reported. It will be important to understand if and how these data may help predict the impact of vaccines in this population and in the older-aged cohort.

## Environmental factors

Ageing is associated with huge metabolic changes which may influence immune responsiveness. In fact, reverse metabolic syndrome is associated with increased mortality in the older adults, while body mass index (BMI) >30 significantly correlates with protection and improved survival.^51^^,^^52^ Obesity is associated with reduced ability to respond to influenza vaccination^53^^,^^54^ and HBV.^55^ In contrast, caloric restriction in animals increases thymic cellularity (CD4+ and CD8+) with increased in vitro responses to antigens.^56^

These effects of caloric restriction appear to be partly mediated by suppression of the mammalian target for rapamycin (mTOR) complex. mTOR inhibitors increase life span in some animal models and improve their ability to respond to BCG and influenza vaccines and improve vaccine efficacy.^57^^,^^58^ More recently, these “immune-potentiating” effects of mTOR inhibitors have been shown in older adults vaccinated against influenza. The exact mechanisms for this remains unknown, although it may be related to the decreased frequency of programmed cell death protein 1 (PD-1)-positive T-cells observed in the subjects after treatment with the mTOR inhibitor.^59^

Alternatively, immunosuppressive drugs can be used in older adults to suppress an effective immune response to infection and/or vaccination. This can include immunosuppressant chemotherapeutic or anti-inflammatory medications including corticosteroids. Moreover, other drugs which are being widely used for comorbidities, such as statins, have been shown to significantly reduce the antibody response to, and clinical effectiveness of, influenza vaccines in older adults.^60^^,^^61^ The precise molecular mechanisms leading to this effect remain elusive, though hypotheses suggest statins may influence the HDL expression on T regulatory cells which would negatively affect the antibody response to specific antigens.^62^ Alternatively, this could be explained by changes induced by statins on cell membrane properties with consequent changes in internal signaling.^63^

It is evident that a comprehensive view has to consider the variable of age in combination with comorbidities which may lead to the deterioration of the body. In particular, frailty is now being taken into consideration when evaluating the susceptibility of aged individuals to infection, their ability to appropriately respond to vaccination, and their risk of poor clinical outcomes after infection. Frailty is a syndrome comprising multiple factors such as physical capability, malnutrition, co-morbidities, cognitive dysfunction, etc., and is strongly associated with ageing, evident in up to 45% of subjects ≥85-year-old.^64^ Some studies have been initiated to define biomarkers associated with various levels of frailty which may predict response to vaccination, In one study, the levels of CD8+ CD27+ CD45RA+ T cells and circulating switched CD19+ memory B cells were significantly decreased in subjects with grade 4 frailty as compared with older subjects without evident frailty.^65^

These findings underline the importance of considering the multifactorial events that are part of immunosenescence, which encompasses not only the intrinsic events associated with the chronological aging of the body, but the environmental factors that contribute to the reduced immune responsive of older adults. Understanding the association between the “intrinsic” and the “extrinsic” factors contributing to reduced responsiveness will enable a better appreciation of the important variability in responses to infections and vaccination in older adults.

## Learning from successes

Considering the pleiotropic nature of immunosenescence and its variable expression among older individuals, it is not surprising that, despite the “physiological” decay of the immune responsiveness with age, vaccination remains a vital intervention in the older adults. Several pharmacoeconomic studies have underlined the benefit of influenza vaccines in terms of lives saved and reduced direct and societal costs linked to reducing influenza-related morbidity and mortality.^66^ Moreover, failure to vaccinate is associated with excess mortality due to infection and its complications.^67^ However, some vaccines have been shown to exhibit sub-optimal efficacy in recipients of advanced age or significant frailty, such as live attenuated vaccine against herpes zoster,^68^ or the conjugated pneumococcal vaccine in subjects ≥75-year-old.^69^ Differences in bio-age, immunobiography and trained immunity can reconcile the apparent discrepancy between the reduced response of the elderly to some existing vaccines, and the evident successes that have been obtained recently. There is reason to believe that we can improve on the former by deciphering the mechanisms underlying the latter.

The efficacy of the seasonal trivalent inactivated influenza vaccine has been estimated to drop significantly in subjects >75-year-old^70^ and even more so during influenza seasons with circulating virus strains exhibiting antigenic drift as compared to the vaccine strains.^71^ It should be noted that immune response against the pandemic H1N1 vaccine was similar between older and young adults,^72^ possibly due to potential imprinting from previous experiences with waves of continually circulating H1N1 virus strains.^73^ This observation supports the hypothesis that it would be “easier” to build on pre-existing immunological memory than inducing *bona fide* novel priming in the older adults. It is not currently possible to determine whether pediatric vaccination would significantly influence the level of immune responsiveness to infection and vaccination at older ages, but this will become a very interesting area of research when the majority of our vaccinated populations reach older ages.

**Fig. 3:**
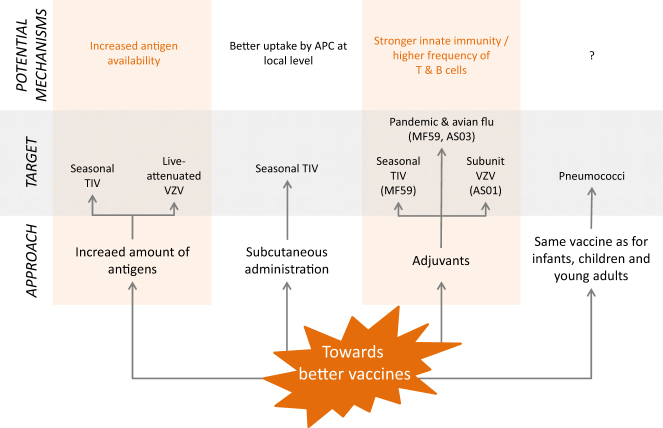
New approaches towards better vaccines for older adults. APC antigen-presenting cell, AS adjuvant system, TIV trivalent influenza vaccine, VZV varicella zoster virus

Three major avenues have been followed so far to improve the performance of vaccines in the older adults: (i) increase the dose of the vaccine (e.g., live attenuated zoster vaccine, TIV, avian flu), (ii) intradermal route of vaccination (e.g., TIV), or (iii) vaccine adjuvants (e.g,. TIV, avian flu, herpes zoster) (Fig. 3).

Increasing the antigen dose significantly enhanced immune responses and improved efficacy to TIV and live-attenuated VZV in phase III trials.^68^^,^^74^ However, this approach gave sub-optimal results when applied to the avian H5N1 influenza vaccine.^75^ These results suggest that higher doses of vaccine antigen may work better in situations where immunological priming already exists and less well in cases when the vaccine is required to prime a de novo response. This is not unexpected, due to the reduced numbers of naïve CD4+ T cells and B cells in older adults compared to fully-immunocompetent younger adults.

Intradermal delivery of vaccines offers the advantage of promoting the migration of vaccine antigens to draining lymph nodes with activation of resident dendritic cells (DC) which can then be induced to migrate. These events involving professional antigen-presenting cells can lead to the activation of antigen-specific T cells and generation of T follicular helper cells in the lymph node, with subsequent activation and affinity maturation of antigen-specific B cells and their maturation into plasma cells. This route of administration allows for significant dose-sparing, as shown by many years of experience with the rabies vaccine.^76^ Nevertheless, it took years, starting from the 1950s, before intradermal administration of TIV was proved successful, particularly in subjects ≥60-years old.^77^ It is important to note that the anatomy and physiology of the skin alter with age, and that the amount and the quality of DC present in the skin of the older adults may differ from those found in healthy young adults.^78^ This implies that not all vaccines will necessarily generate stronger responses when administered intradermally. For example, intradermal vaccination with inactivated H5N1 vaccine gave sub-optimal responses compared to intramuscular vaccines.^79^ Vaccine effectiveness may depend upon the intrinsic characteristics of the vaccine and its interaction with the microenvironment of the injection site. Finally, local reactogenic events are more frequent after intradermal administration than intramuscular administration of TIV.^80^^,^^81^ It is worth noting that these local reactions were observed less frequently in immunocompromised patients, suggesting an immunological mechanism behind these reactions.^82^ Importantly, our knowledge of the immunological events following vaccination in the blood remain unsure, and much larger gaps exist in our knowledge of the events taking place in the skin and at other anatomical sites of older adults.

The field of vaccine adjuvants has recently evolved^83^ following the identification of specific Pathogen-Associated Molecular Pattern (PAMP) receptors on innate cells, e.g., the TLRs, and the identification of microbial or other external components as ligands of these receptors, activators of innate immunity, and eventually as potential vaccine adjuvants. This field has significantly expanded and adapted a mechanistic approach to understanding the function of adjuvants and their appropriate design. Several of these PAMP agonists are now at different stages of development as vaccine adjuvants.^84^ Some, like monophosphoryl lipid A (MPL), a TLR4 agonist, have been combined with other adjuvant components to stimulate specific types of immune response. Together with aluminum salts (in an adjuvant designated AS04) it has been used in subunit vaccines to generate strong antibody and cell-mediated responses against human papillomavirus or hepatitis B virus. These vaccines have been approved for human use and are now widely used.^85^ MPL, together with the saponin A and QS21 in a liposome formulation (an adjuvant system named AS01), has been used in a vaccine against *Plasmodium falciparum* malaria, which has shown efficacy in children in Africa,^86^ and also in a new subunit vaccine against herpes zoster, which is in late phase development.

Herpes zoster occurs following a reactivation of VZV which has established a latent infection in the peripheral nervous ganglia. It can be debilitating and severe condition, and potentially clinically devastating in cases of post-herpetic neuralgia (PHN). Its incidence increases with age and with the decline of immune responsiveness, whether from immunosenescence, immunosuppression, or through infections such as HIV.^12^ The live attenuated vaccine against herpes zoster is efficacious, but its efficacy is lower in older individuals, and wanes rapidly with time post-immunization.^17^

A vaccine able to confer durable protection against herpes zoster and PHN has been made possible using a recombinant protein, gE, the most abundant VZV surface protein, together with the AS01 adjuvant. Two doses of this adjuvanted subunit vaccine were highly immunogenic in phase I and II trials in adults, including older adults, inducing high and persisting levels of specific antibodies and CD4+ T cells.^87–89^ Remarkably, in large scale phase III trials, two doses of this gE-based, adjuvanted subunit vaccine were highly efficacious (>90%) against herpes zoster and efficacy did not differ significantly between >50-year-old and the >70-year-old.^90^ Efficacy was maintained at >90% in subjects >80-year-old, and persisted without significant decline for atleast four years. In addition, vaccination prevented PHN with very high efficacy in vaccinated older cohorts.^91^

The outstanding results of this vaccine appear to be at least partly ascribed to the ability of the AS01 adjuvant to activate in a coordinated manner both the innate and the adaptive immune systems. Results in mice and non-human primates have shown that, following intramuscular injection, AS01 components are rapidly carried to the draining lymph nodes by activated antigen-presenting cells (APCs) and then rapidly eliminated. Induced migration and activation of innate cells (e.g. CD14+ CD16+ monocytes) in the draining lymph nodes occur as early as 24 h post-injection. Remarkably, 4 h post-injection there is detectable production of IFN-γ, evident both by microarray analysis and by quantitation in the serum. It is now known that this early production of IFN-γ is due to NK cells,^92^ which appears to be essential for the activation of dendritic cells and of the development of the Th1-type immunity observed after vaccination.^93^ In summary, the AS01 adjuvant appears to work very strongly at the very early stage post-vaccination on various elements of the innate immunity, those discussed above as important in immunobiography and in trained immunity. Early innate activation may lead to stronger activation of antigen-specific Th1 CD4+ T cells and subsequent activation of B cells capable of producing anti-gE antibodies. Numerous studies are now in progress using a systems vaccinology approach in order to dissect the fine mechanisms triggered by the AS01 adjuvant in older people which lead to this very high and sustained efficacy against herpes zoster and its complications, including PHN.

## Discussion

The progressive aging of the population worldwide poses challenges to health systems in both developed and developing countries. Much has been learned in recent years on the phenomena that characterize the waning of immunological competence that occurs during aging, and on some of the mechanisms underlying the poorer responsiveness of older people to infection and/or vaccination. While vaccine recommendations exist for older adults, performance is often far from optimal, while new vaccines are required to cover diseases that are becoming more and more problematic in aging populations, such as RSV, *Clostridium difficile*, *Staphylococcus aureus* and other antibiotic-resistant bacterial infections.^13^ Based on what has been discussed above, the following key issues need to be addressed:

Basic research is needed to understand the mechanisms behind the immunological events that take place during aging, and particularly the heterogeneity found among the elderly population—the concepts of “immunobiography”^40^ and “bio-age”.^39^ Current research is focused on specific, narrow targets, while a more integrated approach is desirable. Systems vaccinology approaches could shed light on multiple parameters at the same time and define possible biomarkers of vaccine efficacy or of vaccine hyporesponsiveness.^39^^,^^42^

There is a need for more translational research in older populations, including systems vaccinology to validate new scientific findings and novel technologies suitable for vaccine development. Not all findings in healthy adults are necessarily transferable to older adults. Even the most recent examples of vaccination success in older adults, were derived from mechanistic knowledge gained from younger immunocompetent individuals, either humans or mice. Proof-of-principle studies conducted in older cohorts, including parameters of frailty, would improve our ability to optimize new vaccines to counteract the decline of immune competence in older adults. As discussed above, adjuvants such as AS01 can strongly activate elements of innate (and possibly trained)^93^ immunity. These effects may underlie the strong, long-lasting efficacy demonstrated against herpes zoster even in very old subjects.^92^

Understanding how older individuals differ from each other not only based on their genetic make-up, but also on their environment and history of previous infections/diseases, diet, physical activity, co-morbidities and pharmacological treatments (their immunobiography)^40^ can influence the outcome of the immunological analyses we do. For example, most of the information on the immune responsiveness of older adults to vaccines comes from studies with seasonal TIV, a vaccine they may receive every year, against a virus that they have encountered frequently since their childhood. This continuous exposure can, on one side, help establish a sizeable memory able to respond to subsequent encounters with the virus and/or the vaccine, as suggested by recent reports.^73^ However, this memory could provide a form of “imprinting” or trained immunity^41^ which may further reduce the ability to respond to novel virus variants. The same could be said for other disease models.

Finally, most of what we know about the immune response to vaccination comes from blood samples which are the easiest biological samples to obtain. However, little is known regarding the phenomena taking place at priming sites (muscle, skin, regional lymph nodes) or effector sites, such as the respiratory mucosa. Changes at these anatomical sites can be expected to significantly affect the priming and effector arms of the immune response induced by vaccines given to the older adults.^78^^,^^94^ The precise role played by these changes requires further research to gain a full understanding of the mechanisms involved.

In conclusion, an integrated approach to better understand aging and health and how vaccines can help overcome some of the issues of immunosenescence is the basis for allowing populations to achieve healthy aging and better quality of life.

### Data availability statement

All data here disclosed are published in the literature as indicated in the references section.
